# The Genome-Wide Analysis of Carcinoembryonic Antigen Signaling by Colorectal Cancer Cells Using RNA Sequencing

**DOI:** 10.1371/journal.pone.0161256

**Published:** 2016-09-01

**Authors:** Olga Bajenova, Anna Gorbunova, Igor Evsyukov, Michael Rayko, Svetlana Gapon, Ekaterina Bozhokina, Alexander Shishkin, Stephen J. O’Brien

**Affiliations:** 1 Theodosius Dobzhansky Center for Genome Bioinformatics, St. Petersburg State University, St. Petersburg, Russia; 2 Department of Genetics and Biotechnology, St. Petersburg State University, St. Petersburg, Russia; 3 Institute of Cytology RAS, St. Petersburg, Russia; 4 Boston Children’s Hospital, Boston, MA, United States of America; 5 Division of Biology and Biological Engineering, California Institute of Technology, Pasadena, California, United States of America; 6 Oceanographic Center, 8000 N. Ocean Drive, Nova Southeastern University, Ft Lauderdale, Florida, 33004, United States of America; Swedish Neuroscience Institute, UNITED STATES

## Abstract

Сarcinoembryonic antigen (CEA, CEACAM5, CD66) is a promoter of metastasis in epithelial cancers that is widely used as a prognostic clinical marker of metastasis. The aim of this study is to identify the network of genes that are associated with CEA-induced colorectal cancer liver metastasis. We compared the genome-wide transcriptomic profiles of CEA positive (MIP101 clone 8) and CEA negative (MIP 101) colorectal cancer cell lines with different metastatic potential *in vivo*. The CEA-producing cells displayed quantitative changes in the level of expression for 100 genes (over-expressed or down-regulated). They were confirmed by quantitative RT-PCR. The KEGG pathway analysis identified 4 significantly enriched pathways: cytokine-cytokine receptor interaction, MAPK signaling pathway, TGF-beta signaling pathway and pyrimidine metabolism. Our results suggest that CEA production by colorectal cancer cells triggers colorectal cancer progression by inducing the epithelial- mesenchymal transition, increasing tumor cell invasiveness into the surrounding tissues and suppressing stress and apoptotic signaling. The novel gene expression distinctions establish the relationships between the existing cancer markers and implicate new potential biomarkers for colorectal cancer hepatic metastasis.

## Introduction

Intestinal cancers rank fourth in cancer incidence in the world. Despite the improvement of early diagnostics, 20% of primary colorectal cancer diagnoses reveal remote metastases. Available therapies still offer a poor prognosis and patients have a less than 10% five year survival rate [1]. The serum CEA test is recommended by the American Society of Clinical Oncology [2] and by the European Group on Tumor Markers [3] as a prognostic and postoperative marker for metastases and as an aid in the management of cancer patients. Clinical efficacy of CEA screening has been demonstrated in the follow-up management of patients with colorectal, breast, lung, prostate, pancreatic and ovarian carcinoma [4].

CEA is a large glycoprotein (~180 kD), a member of the *Carcinoembryonic antigen* gene family and the immunoglobulin (Ig) gene superfamily and comprises an exceptionally diverse array of highly glycosylated glycoproteins http://www.carcinoembryonic-antigen.de/index.html [5]. *CEACAM* genes are expressed in multiple cell types including epithelial, endothelial and immune cells such as leukocytes and dendritic cells. CEACAM molecules are generally inserted into the cell membrane via a transmembrane domain or physically linked to membrane via glycosyl-phosphatidylinositol anchorage [5]. Regulation of intercellular adhesion is a major function of CEA [6] and CEA can establish and maintain tissue architecture and function in the colon. The tumorigenic effects of CEA include inhibiting cell differentiation, blocking cell polarization, distorting tissue architecture and inhibiting anoikis (cell death due to the loss of cell-cell contacts) [7, 8]. Nonetheless, the molecular mechanism of CEA related metastasis is not well understood.

We used 2 human colorectal derived MIP101 cell lines of the same origin with a different metastatic potential [9] to study the influence of CEA on metastasis. Original low-differentiated, poorly metastatic MIP101 cell lines do not produce CEA. The derivative MIP101-clone 8 was genetically modified by transfection with a construct contaning the full-length *CEA* gene and a G418 antibiotic resistance selected to express CEA.

Here we measured transcriptome differences induced by CEA production in colorectal cancer cells with differing levels of CEA production and metastatic potential. The RNA sequencing technology (RNAseq) allows for the comparison of RNA produced by different cell lines, estimation of the level of gene expression, and identification of changes in gene splicing and in the signaling pathways that are involved in response to CEA over-expression.

## Materials and Methods

### Cell Culture

MIP101 is the CEA-negative cell line and MIP 101 clone 8 is the CEA-positive cell line. Both cell lines are generous gift from Dr. P. Thomas of Creighton University, Omaha, NE. The MIP 101 clone 8 cell line was produced by transfection of MIP-101 with the full-length CEA cDNA and was selected by culturing the cells in the presence of G 418 (Thermo Scientific, Lafayette, CA, USA). The cell lines were cultured under the same conditions in a sterile incubator at 37 degrees C in a liquid RPMI (Invitrogen Life Technologies, CA) medium with supplements on Petri dishes until 70–80% of the confluence. The RPMI 1640 (Invitrogen Life Technologies, CA) medium was supplemented with 10% fetal bovine serum (FBS; Hyclone, Thermo Scientific, Lafayette, CA, USA), 100tte, CA, USAA,ntifiSigma-Aldrich, St. Louis, MO, USA), 100Aldrich, St. Louis, MSigma-Aldrich, St. Louis, MO, USA), and 300ich, St. Louis, MOSigma-Aldrich, St. Louis, MO, USA) at 37ldrich, St. Lo_2_ atmosphere. Cells were trypsinized and counted. Over 10 million cells from each strain were collected by centrifugation. Two cell lines were cultured in two replicates.

### RNA Isolation and Sequencing

Total RNA was extracted from human colon carcinoma cells using Trizol reagent, according to the manufacturer's protocol (Invitrogen Life Technologies, CA) and cleared from ribosomal RNA. Poly-(A)RNA were isolated from 3 mg of total RNA using Sera-Mag oligo (dT) spheres (Thermo Scientific, Lafayette, CA, USA).

Libraries for sequencing were obtained using the Truseq kit, universal adapter sequences, and specific PCR primers, recommended by Illumina. (Illumina, SanDiego, CA, USA). The mRNA sequencing libraries were prepared and sequenced using an Illumina HiSeq2000 instrument at the Broad Institute, Boston, USA. More than 50 M reads were produced for each library with read length of 76 bp.

### Transcriptome analysis

We tested sequence reads using the quality control program FastQC (http://www.bioinformatics.babraham.ac.uk/projects/fastqc/) [10]. Reads were cleaned using FASTX-Toolkit (http://hannonlab.cshl.edu/fastx_toolkit/) to remove the low-quality reads and traces of Illumina adapter sequences.

Clean reads were mapped to the GRCh37 human reference genome using TopHat (v2.0.9) as part of a Tuxedo pipeline [11]. The expression level for the genes and their isoforms were calculated by Cufflinks and resulted in FPKM values (fragments per kilobase of exon per million fragments mapped). Differential expression levels between samples were calculated by EdgeR package [12], and the p-value was adjusted using the FDR (false discovery rate) control method. EdgeR package is based on the statistical method quantile-adjusted conditional maximum likelihood estimator for the dispersion parameter of the negative binomial distribution which was developed for very small sample sizes, typical of those from analysis of gene expression studies with small number of observations (e.g. 2 libraries for a given experimental condition) [12]. The genes were considered differentially expressed between two conditions if they showed a log2 fold-change ratio more than 2 and displayed an FDR-adjusted p-value <0.05. We identified relevant biological processes and molecular pathways using the Gene Set Enrichment Analysis (GSEA) tool (http://www.broadinstitute.org/gsea).

### Validation of RNA-sequencing data by qRT-PCR

Using the Primer 3 program (http://www.ncbi.nlm.nih.gov/tools/primer-blast/) PCR primers were designed to be suitable for all significant isoforms of each differentially expressed gene; forward and reverse primers were located in different exons of the corresponding gene (S3 Table). The sequences of the selected primers shown in the S3 Table were used, the expression levels of the *CEA* and *GAPDH* genes were used as controls for PCR analysis. Each qRT-PCR reaction was performed with three replicates.

## Results

### Global Gene Expression Profiling by RNA sequencing

**Fig 1:**
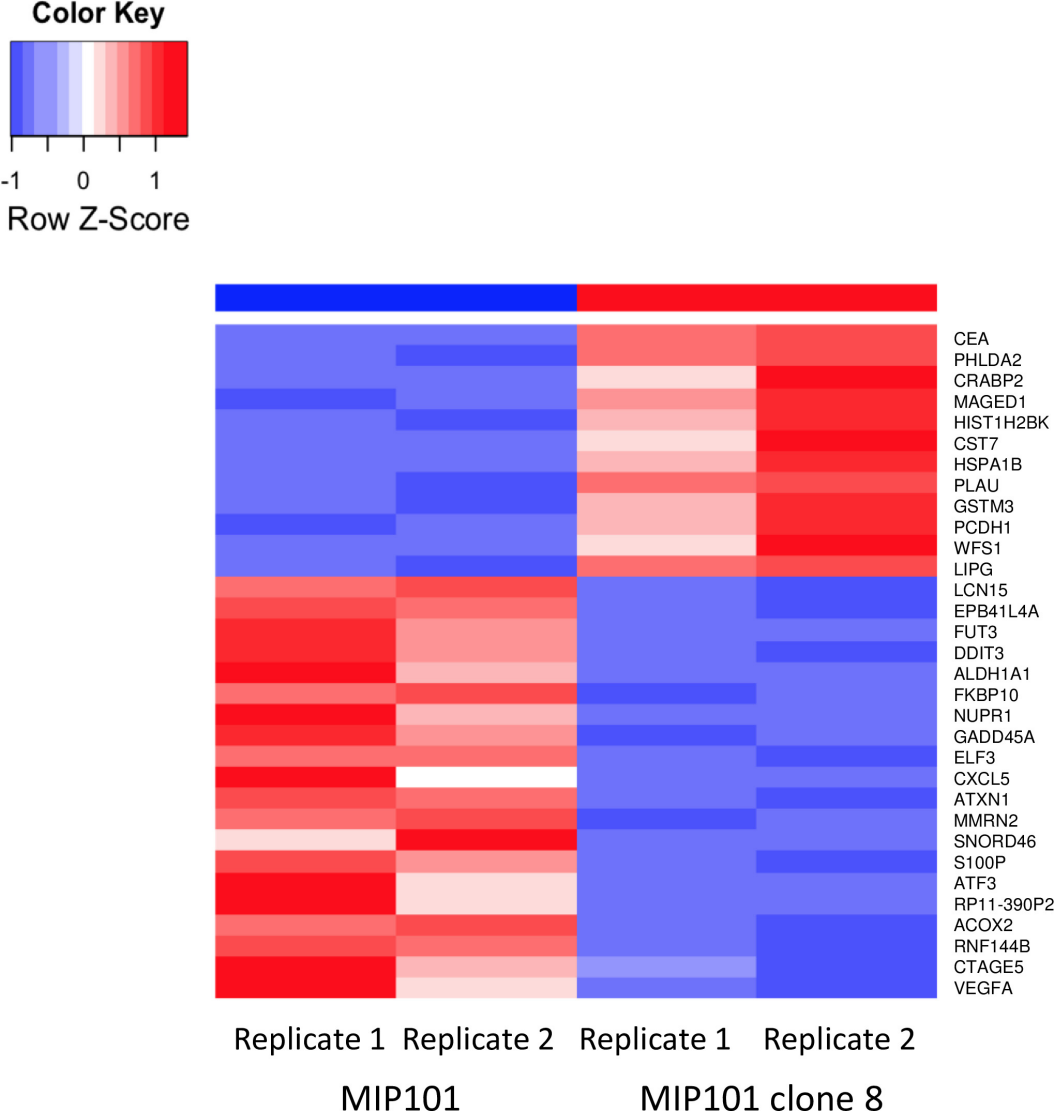
The top 30 upregulated and downregulated genes in CEA+ and CEA- cells. The red color indicates upregulated mRNA; the blue color indicates downregulated mRNA. On the right side of the square are the names of the genes that differ in the expression between MIP101 (CEA-) and MIP101 clone 8 (CEA +) cell lines. The experiments were performed in two replicates starting from RNA extraction. As a control, we performed an analysis of the *CEA* gene and its isoforms expression level in these cell lines by RNA-seq data. Fig 2 shows plots of the *CEA* expression. Expression of the *CEA* gene is present in the CEA-producing line (MIP101 clone 8) and absent in the CEA-deficient cell line (MIP101). Moreover, CEA gene was represented by one isoform *CEACAM5-001* (Fig 2B).

To analyze the changes of transcriptome associated with CEA up-regulation, we compared global transcript levels in the CEA-up-regulated (MIP101 clone 8) and CEA negative (MIP101) cell lines using the Tophat pipeline [10, 11]. One hundred transcripts of known genes were significantly different in expression. 32 genes were up-regulated and 68 were down-regulated in CEA-producing cells having a fold change in expression >2 and FDR < 0.05 (S1 Table). The top 30 up-regulated and down-regulated genes with an FDR < 0.0001 are summarized in Fig 1.

**Fig 2:**
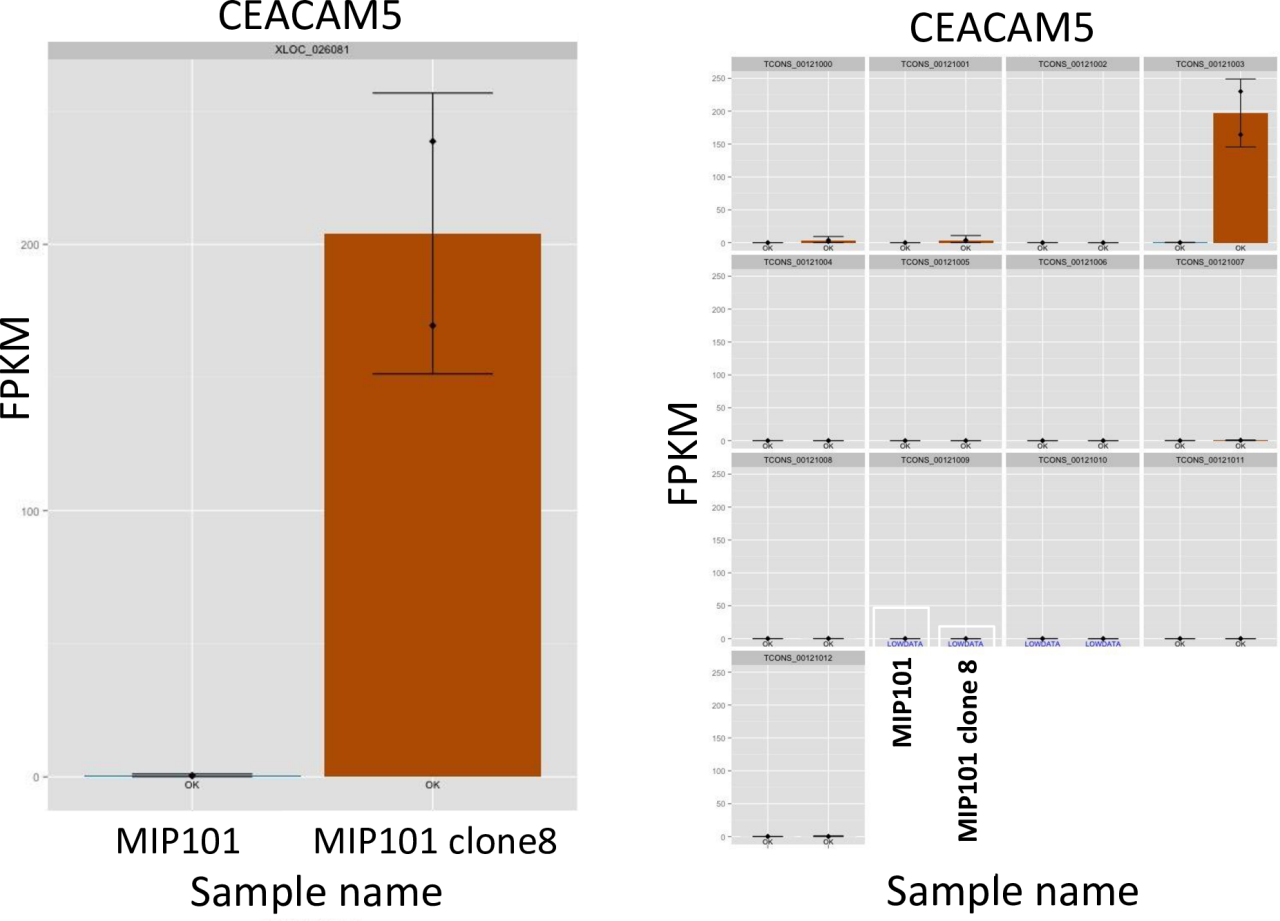
Differential expression analysis results for *CEA (CEACAM5)* gene. a. Expression plot shows differences in the *CEACAM5* expression level between MIP101 (CEA-, blue color) and MIP101 clone 8 (CEA+, brown color) cell lines, measured in FPKM. Each sample was represented by two replicates. b. Expression level of *CEACAM5* is represented by single *CEACAM5-001* isoform.

### Molecular Pathways and Biological Processes Analysis

In order to identify putative biological processes affected by changes in gene expression, we performed Gene Ontology enrichment analysis using the Gene Set Enrichment Analysis (GSEA) tool (http://www.broadinstitute.org/gsea). 49 biological processes were functionally enriched including cell proliferation and apoptosis in response to stress and other cancer-related processes. The top 10 identified biological processes are summarized in Table 1 and the complete list is presented in the S2 Table.

**Table 1:** Top 10 functional enriched GO biological processes regulated by CEA expression level in MIP101 colorectal cancer cells.

Gene ontology term ID	Term name	P-value		FDR q-value
GO:0008283	Cell proliferation		5.67E-09	2.20E-06
GO:0006915	Apoptosis		1.87E-07	2.47E-05
GO:0006950	Response to stress		7.40E-06	5.09E-04
GO:0008544	Epidermis development		1.21E-05	7.67E-04
GO:0007398	Ectoderm development		1.94E-05	1.14E-03
GO:0050793	Regulation of developmental process		2.65E-05	1.46E-03
GO:0007165	Signal transduction		8.28E-05	4.02E-03
GO:0006350	Transcription		1.19E-04	5.46E-03
GO:0009888	Tissue development		1.63E-04	6.37E-03
GO:0006139	Nucleotide and nucleic acid metabolic process		1.64E-04	6.37E-03

Molecular pathways regulated by CEA over-expression were identified by pathway analysis using Kyoto Encyclopedia of Genes and Genomes (KEGG). In total, we identified 4 significantly overrepresented pathways: cytokine-cytokine receptor interaction, MAPK signaling pathway, TGF-beta signaling pathway and pyrimidine metabolism (see Table 2).

**Table 2:** Molecular pathway analysis of genes regulated by CEA expression level in MIP101 cells by KEGG database.

Description	P-value	FDR q-value
Cytokine-cytokine receptor interaction	1.50E-05	1.40E-03
MAPK signaling pathway	1.50E-05	1.40E-03
TGF-beta signaling pathway	6.61E-04	4.10E-02
Pyrimidine metabolism	9.66E-04	4.49E-02

Phenotypic changes in CEA over-expression cells MIP101 clone 8 may be caused by alterations in expression levels of genes, associated with cancer progression and metastasis. Table 3 summarizes phenotype-related genes which were selected from the list of differently expressed genes after CEA over-expression.

**Table 3:** Phenotype-related genes in MIP101 clone 8 cells with CEA over-expression.

Gene name	Description	Log2 fold change	P-value	FDR
***TGFB2***	transforming growth factor, beta 2	2.09	1.14E-04	1.38E-02
***VEGFA***	vascular endothelial growth factor A	-2.08	1.15E-06	2.69E-04
***CXCL5***	chemokine (C-X-C motif) ligand 5	-8.87	2.27E-11	1.59E-08
***TNFSF15***	tumor necrosis factor (ligand) superfamily, member 15	-2.15	1.60E-04	1.80E-02
***FGF21***	fibroblast growth factor 21	-4.18	2.38E-06	5.20E-04
***GADD45A***	growth arrest and DNA-damage-inducible, alpha	-2.28	1.60E-13	1.52E-10
***HSPA1B***	heat shock 70kDa protein 1B	3.24	3.06E-10	1.75E-07
***MAP2K6***	mitogen-activated protein kinase kinase 6	-3.75	1.70E-04	1.90E-02
***MAGED1***	melanoma antigen family D, 1	2.07	1.14E-11	8.43E-09
***KLF11***	Kruppel-like factor 11	-2.61	3.25E-05	4.77E-03

### Validation of RNA-seq data by qRT-PCR

We performed qRT-PCR to validate RNA-seq technology and bioinformatics methods. Three up-regulated genes (*DSP*, *PCDH1* and *WFS1*) and two down-regulated genes (*GADD45A* and *KLF11*) were randomly selected from the list of differently expressed genes.

**Fig 3:**
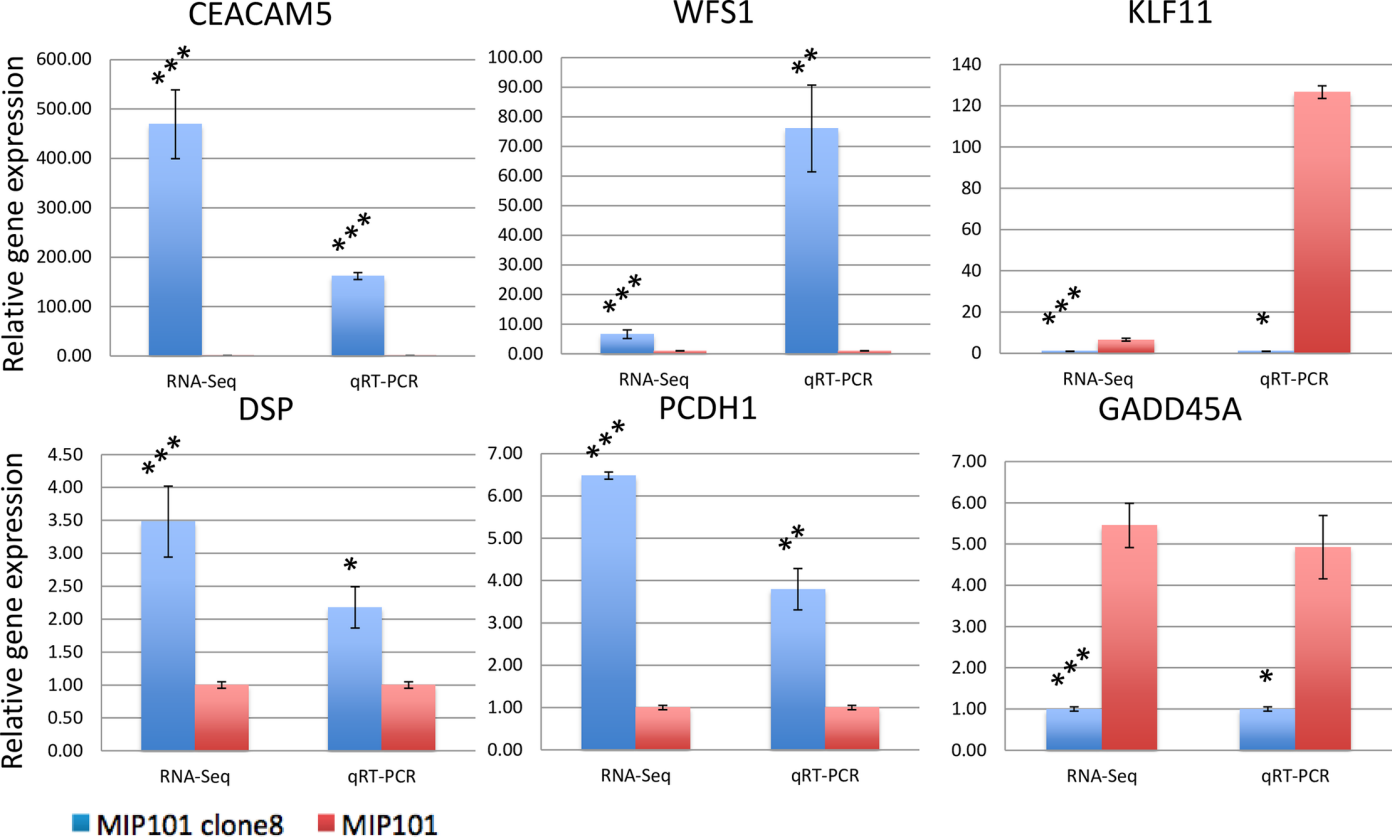
Validation of RNA-Seq results was performed by qRT-PCR analysis on 5 randomly chosen genes from the list of differentially expressed genes. CEACAM5 also was analysed as positive control. Results obtained by RNA-Seq and qRT-PCR methods show high accordance. Each qRT-PCR was performed with three replicates. All data are means ± SD. (*p < 0.05; **p < 0.01, ***p < 0.001, one-way ANOVA for qRT-PCR and FDR-adjusted exact test for RNA-Seq).

*CEA* gene expression was used as a positive control and *GAPDH* gene was used as an endogenous control. As demonstrated in Fig 3, the up-regulation and down-regulation of all selected genes were in accordance with RNA-seq analysis results. The results of classic PCR analysis are presented in S1 Fig.

## Discussion

The steady growth of cancer incidence, increasing survival ages, and high mortality from cancer metastasis requires the development of more specific and effective methods for diagnostics and prevention of metastasis. Metastasis is the end product of a multi-step, cell-biological process termed the invasion-metastasis cascade which involves dissemination of cancer cells to anatomically distant organs and their subsequent adaptation to a foreign tissue microenvironment. Each of these events is driven by the acquisition of genetic and epigenetic alterations within the tumor cells and multiple interactions between the metastatic population and microenvironment [13]. Metastasis is a highly specific process. Colorectal cancer cells frequently develop metastases to the liver and lungs, whereas prostate and breast cancers more frequently target the bones as their site for metastasis [13].

Although CEA plays a key role in the formation of hepatic metastasis from colorectal cancer the precise mechanism for CEA-induced metastasis is not yet well understood [14]. We discovered and cloned the gene for the CEA receptor from the liver macrophages—Kupffer cells [15]. It has been shown that the CEA/CEAR interaction on the surface of Kupffer cells stimulates the IL1a, IL6, IL10 and TNF-alpha cytokine production and influences the cell adhesion molecules in the liver that enhances the survival and homing of metastatic cancer cells in the liver that trigger metastasis [16].

In this study we carried out an in-depth analysis on the effect of CEA on colorectal cancer cells. The new information on the gene expression, biological processes and signaling pathways involved in response to CEA were elucidated. The transcriptomic profiling revealed changes in the level of expression for 100 genes (Fig 1) including *VEGFA*, *CXCL5*, *TNFSF15*, *PCDH1 etc*.

Previously, we have shown the influence of CEA synthesized by cancer cells on the formation and function of E-cadherin adhesion junction complexes [8]. These complexes are one of the main contacts between the epithelial cells. CEA disrupts the interactions between the proteins that form these complexes: E-cadherin, alpha- and beta-catenins, and catenin p120. CEA production also changed the splicing variants of catenin p120 protein and triggered the nuclear expression of beta-catenin [8]. P120-catenin is a master regulator of cadherin stability and a modulator of Rho GTPase activity. Although p120-catenin stabilizes E-cadherin at the plasma membrane and promotes cell-cell adhesion, it can also promote cell motility and invasion [17].

Using the Gene Ontology (GO) enrichment analysis we discovered 49 GO biological processes that were functionally enriched by CEA including cell proliferation, apoptosis, response to stress, transcription and nucleic acid metabolism (Table 1). First, CEA receptor protein (CEAR) has been described as heterogeneous nuclear RNA-binding protein M (HNRNPM1-4) [18]. This protein belongs to a large family of heterogeneous nuclear RNA-binding proteins (HNRNPs A-U), also called “histones of RNA” [18]. It is an abundant nuclear shuttling protein that has at least 4 protein isoforms [18] and only 2 transcripts have been experimentally validated: the full-length protein (isoform 1) and the short form (isoform 2) that has a deletion [19]. We identified the isoform 2 as the CEA-binding protein on the surface of Kupffer cells [20].

The HNRNPM is a multifunctional protein that is involved in mRNA processing [21] and splicing [22]. In human cells, CEAR/ HNRNPM plays a role in regulating FGFR2 alternative splicing and can affect the splicing of several other genes [23].

Post-transcriptional modifications can modulate HNRNPM activity by altering its localization, its RNA binding specificity, and interaction with other cellular factors [19]. Generally, HNRNPM has a diffuse nuclear distribution and remains bound to the mRNA as it is transported through nuclear pores. Using isoform specific antibodies, we have shown that in macrophages and colorectal cancer cells the full-length HNRNPM protein-isoform 1 is localized in the nucleus versus the short isoform 2, which appears in the cytoplasm and on the surface of the cells [20].

In support of our data, new evidence has emerged regarding CEA/CEAR signaling pathway activation in the endothelial cells [24]. Endothelial cells do not synthesize CEA. However, soluble CEA, produced by cancer cells, binds with the CEAR receptor on the endothelial cells. Importantly, down-regulation of HNRNPM /CEAR and not VEGF in endothelial cells suppresses tumor angiogenesis [24]. Clinical research also shown that up-regulation of HNRNPM/CEAR protein is associated with the aggressive types of colon [25] and breast cancers [22]. Together, these results elucidate a novel function for CEA/CEAR signaling in the tumor angiogenesis and metastasis.

Using RNA sequencing we performed KEGG analysis and identified 4 new significantly enriched by CEA over-expression pathways: cytokine-cytokine receptor interaction, MAPK signaling pathway, TGF-beta signaling pathway and pyrimidine metabolism (see Table 2).

The transforming growth factor-β (TGF-β) signaling pathway is involved in the control of multiple cancer-related biological processes, including cell proliferation, differentiation, migration, and apoptosis. TGF-β and CEA signaling also induce the epithelial-mesenchymal transition that is essential for metastasis in tumor cells. Recently it has been reported that TGF-β may signal through HNRNPM/CEAR [26]. The authors suggested that NF-kappaB could be a common point between these pathways [26]. Our data also show that MAPK pathway is deregulated by CEA overexpression. MAPK signaling pathway is closely related to the TGF-β signaling pathway.

In this study the large-scale RNA sequencing analysis has provided a wealth of information on the biologically relevant systems that are involved in the response to CEA. We identified the key signaling pathways and proteins that are involved in the CEA-induced colorectal cancer. It represents a major step toward the understanding of the mechanism of colorectal cancer hepatic metastasis.

A successful design of novel therapeutic regimens to target CEA producing cancers is only possible if the network-based pathways are correctly identified. Uncovering the underlying molecular mechanisms of CEA/CEAR signaling in angiogenesis and metastasis will lead to the development of new therapeutic possibilities and agents to suppress the CEA expression and to prevent metastasis.

## Supporting Information

S1 Fig (DOCX)

S1 Table (DOCX)

S2 Table (DOCX)

S3 Table (DOCX)
