# Supplementary material for: The Genome-Wide Analysis of Carcinoembryonic Antigen Signaling by Colorectal Cancer Cells Using RNA Sequencing
Source: PLoS One. 2016 Sep 1;11(9):e0161256. doi: 10.1371/journal.pone.0161256 (PMC5008809; doi:10.1371/journal.pone.0161256)
Supplement: S1 Fig — (DOCX) [file pone.0161256.s001.docx]

**S1_Fig.pdf Validation of RNA-Seq results by RT-PCR.**

| 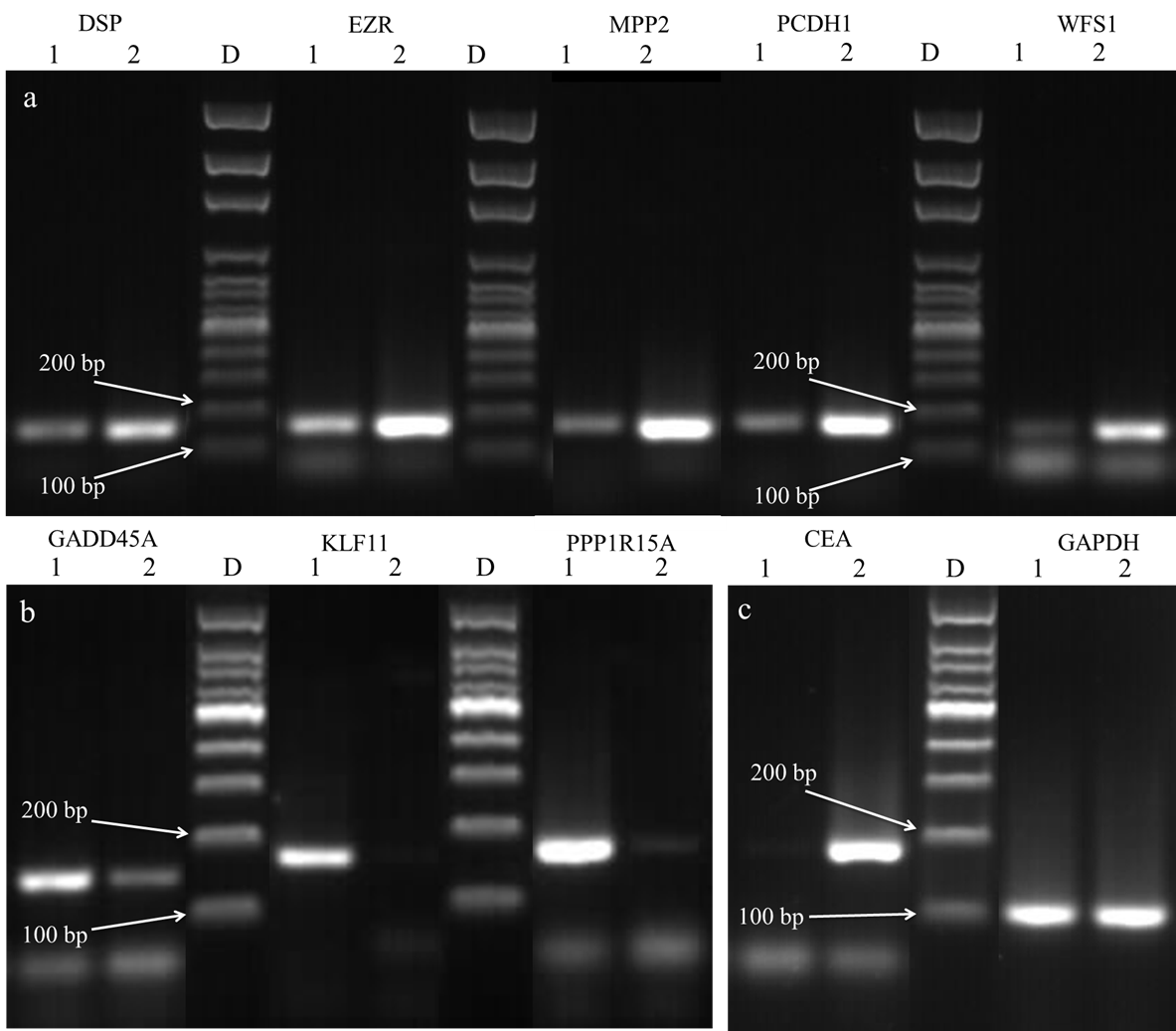 |
| --- |
| Line 1 represents RT-PCR products for MIP101 cell line.  Line 2 - represents RT-PCR products for MIP101 clone 8 cell line.  **a.** RT-PCR products for genes that are over-expressed in CEA-producing (MIP101 clone 8) cell line.  **b.** RT-PCR products for genes down-regulated in the CEA-producing cells.  **c.** RT-PCR products for control genes: *CEA* gene expression is absent in MIP101 cell line, *GAPDH* genes are equally expressed in CEA-producing and deficient cell lines. Both cell lines were represented by two replicates. |
