# Supplementary material for: The Genome-Wide Analysis of Carcinoembryonic Antigen Signaling by Colorectal Cancer Cells Using RNA Sequencing
Source: PLoS One. 2016 Sep 1;11(9):e0161256. doi: 10.1371/journal.pone.0161256 (PMC5008809; doi:10.1371/journal.pone.0161256)
Supplement: S1 Table — (DOCX) [file pone.0161256.s002.docx]

**S1_Table.xls.** **The complete list of differently expressed genes in MIP101 clone 8 cells with CEA overexpression**

| Gene name | Locus | Log2 fold change | PValue | FDR |
| --- | --- | --- | --- | --- |
| *CEA,CEACAM5,CEACAM6* | 19:42212503-42290801 | 8.26 | 3.77E-69 | 5.88E-65 |
| *LCN15* | 9:139654085-139660707 | -4.61 | 1.65E-37 | 1.03E-33 |
| *EPB41L4A-AS1* | 5:111478137-111755013 | -2.23 | 1.59E-28 | 6.18E-25 |
| *PHLDA2* | 11:2949502-2950685 | 2.10 | 2.29E-18 | 3.76E-15 |
| *FUT3* | 19:5842898-5858250 | -5.90 | 5.32E-18 | 8.09E-15 |
| *DDIT3,MIR616* | 12:57828526-57941114 | -2.19 | 6.10E-18 | 8.64E-15 |
| *ALDH1A1* | 9:75515577-75695358 | -7.72 | 2.21E-16 | 2.71E-13 |
| *FKBP10* | 17:39958198-39979465 | -4.40 | 5.07E-16 | 6.08E-13 |
| *CRABP2,RP11-66D17.5* | 1:156669397-156682966 | 5.90 | 4.98E-15 | 5.54E-12 |
| *NUPR1* | 16:28548605-28550495 | -5.62 | 1.14E-14 | 1.22E-11 |
| *GADD45A* | 1:68150743-68154021 | -2.28 | 1.60E-13 | 1.52E-10 |
| *ELF3,RP11-510N19.5* | 1:201951499-201986316 | -2.22 | 5.04E-12 | 3.83E-09 |
| *MAGED1* | X:51546102-51645453 | 2.07 | 1.14E-11 | 8.43E-09 |
| *HIST1H2BK* | 6:27104753-27114619 | 2.09 | 1.67E-11 | 1.21E-08 |
| *CXCL5* | 4:74861358-74864496 | -8.87 | 2.27E-11 | 1.59E-08 |
| *CTD-2373H9.6,TIMP2* | 17:76849012-76921469 | 3.65 | 1.85E-10 | 1.16E-07 |
| *CST7* | 20:24929865-24940564 | 5.02 | 2.03E-10 | 1.23E-07 |
| *HSPA1B* | 6:31789925-31798031 | 3.24 | 3.06E-10 | 1.75E-07 |
| *KLK11* | 19:51525471-51531295 | -5.78 | 4.78E-10 | 2.55E-07 |
| *PLAU* | 10:75668934-75682535 | 4.41 | 1.70E-09 | 8.15E-07 |
| *ATXN1* | 6:16299177-16771317 | -2.07 | 2.78E-09 | 1.25E-06 |
| *RP11-48O20.4* | 1:159930960-159957526 | -3.87 | 3.90E-09 | 1.73E-06 |
| *MMRN2,RP11-96C23.8* | 10:88695296-88784489 | -2.36 | 5.59E-09 | 2.39E-06 |
| *RB1CC1* | 8:53535015-53658403 | 6.88 | 6.18E-09 | 2.53E-06 |
| *SNORD46* | 1:45205489-45248029 | -7.47 | 8.71E-09 | 3.49E-06 |
| *S100P* | 4:6683178-6698904 | -2.41 | 9.99E-09 | 3.94E-06 |
| *GSTM3* | 1:110198702-110318050 | 2.04 | 1.54E-08 | 5.85E-06 |
| *ATF3* | 1:212738675-212794298 | -2.26 | 3.86E-08 | 1.37E-05 |
| *PCDH1* | 5:141230167-141258811 | 2.64 | 6.88E-08 | 2.28E-05 |
| *RP11-125B21.2* | 9:2421602-2656221 | -3.21 | 2.03E-07 | 5.88E-05 |
| *WFS1* | 4:6271575-6305108 | 2.66 | 2.13E-07 | 6.09E-05 |
| *RP11-390P2.4* | 6:138736176-139018768 | -2.47 | 3.17E-07 | 8.71E-05 |
| *SEZ6L2* | 16:29882479-29910984 | -4.95 | 3.24E-07 | 8.82E-05 |
| *RNY4* | 7:148660406-148660502 | -9.94 | 3.64E-07 | 9.77E-05 |
| *LIPG* | 18:47086771-47119276 | 2.57 | 3.97E-07 | 1.04E-04 |
| *ACOX2* | 3:58490862-58523046 | -3.23 | 5.62E-07 | 1.40E-04 |
| *RNF144B* | 6:18366966-18469108 | -3.10 | 5.76E-07 | 1.43E-04 |
| *CTAGE5,MIA2* | 14:39699434-39856306 | -2.20 | 7.30E-07 | 1.79E-04 |
| *VEGFA* | 6:43737920-43754224 | -2.08 | 1.15E-06 | 2.69E-04 |
| *FSCN1* | 7:5632438-5646335 | 4.43 | 1.18E-06 | 2.76E-04 |
| *CAPG* | 2:85620941-85645555 | -2.71 | 1.35E-06 | 3.12E-04 |
| *RP11-328L11.1* | 8:96216683-96822364 | 9.44 | 1.46E-06 | 3.33E-04 |
| *MIR1225* | 16:2089815-2185899 | -9.42 | 1.53E-06 | 3.46E-04 |
| *RP11-400N13.2* | 1:222054322-222159350 | 2.09 | 1.56E-06 | 3.52E-04 |
| *FGF21* | 19:49223842-49261587 | -4.18 | 2.38E-06 | 5.20E-04 |
| *ZFP57* | 6:29640168-29648941 | 4.78 | 2.48E-06 | 5.34E-04 |
| *SEMA6B* | 19:4542550-4581503 | 3.85 | 2.48E-06 | 5.34E-04 |
| *SNORD14A* | 11:17095929-17229536 | -9.22 | 2.62E-06 | 5.55E-04 |
| *EFHD1* | 2:233412778-233547491 | -4.68 | 4.07E-06 | 8.18E-04 |
| *SNORA72* | 8:98881067-99058697 | -6.23 | 4.85E-06 | 9.57E-04 |
| *C15orf48,HMGN2P46,MIR147B,RP11-519G16.3* | 15:45722726-45983492 | -2.22 | 6.79E-06 | 1.26E-03 |
| *UNC5B* | 10:72972326-73062621 | -3.11 | 1.07E-05 | 1.84E-03 |
| *LY6G6D,LY6G6F,MEGT1* | 6:31654701-31685695 | -2.89 | 1.23E-05 | 2.06E-03 |
| *DHRS3* | 1:12627938-12677737 | -2.47 | 1.27E-05 | 2.11E-03 |
| *TIMP4* | 3:12045861-12232907 | -2.42 | 1.49E-05 | 2.43E-03 |
| *ADM2* | 22:50919984-50924919 | -5.90 | 1.63E-05 | 2.62E-03 |
| *SLC16A4* | 1:110905469-110974982 | -2.04 | 2.30E-05 | 3.51E-03 |
| *RNY4P20* | 6:151561133-151679692 | 8.36 | 2.62E-05 | 3.93E-03 |
| *FRMD3* | 9:85857904-86153461 | -2.01 | 2.62E-05 | 3.93E-03 |
| *KLF11* | 2:10170757-10195011 | -2.61 | 3.25E-05 | 4.77E-03 |
| *PRSS1,PRSS3P2* | 7:142457222-142482416 | -4.24 | 3.57E-05 | 5.19E-03 |
| *CBR1* | 21:37402367-37498938 | 5.99 | 3.94E-05 | 5.64E-03 |
| *GPR1* | 2:207040039-207082771 | -2.91 | 4.21E-05 | 5.94E-03 |
| *FAM129A* | 1:184759857-184943682 | -4.59 | 4.57E-05 | 6.31E-03 |
| *C1orf85* | 1:156252725-156265463 | -2.61 | 4.70E-05 | 6.47E-03 |
| *FAM107B* | 10:14560553-14816896 | 2.76 | 4.82E-05 | 6.62E-03 |
| *COCH* | 14:31343719-31565747 | -4.33 | 4.94E-05 | 6.76E-03 |
| *RP11-134G8.6,RP11-134G8.7,RPS10P7* | 1:201452657-201504084 | -2.73 | 5.60E-05 | 7.50E-03 |
| *MIR663A,RP3-410C9.1* | 20:26167555-26232162 | 6.05 | 6.80E-05 | 8.92E-03 |
| *LDLRAD4* | 18:13217496-13652754 | -4.87 | 7.18E-05 | 9.33E-03 |
| *A2MP1,RP11-118B22.4* | 12:9381002-9428413 | -4.93 | 7.17E-05 | 9.33E-03 |
| *RGS16* | 1:182567757-182580289 | -2.78 | 7.38E-05 | 9.57E-03 |
| *GLI1,INHBC,INHBE* | 12:57828526-57941114 | -3.66 | 9.69E-05 | 1.20E-02 |
| *TGFB2* | 1:218517537-218619101 | 2.09 | 1.14E-04 | 1.38E-02 |
| *C10orf91* | 10:134243205-134262912 | -2.51 | 1.35E-04 | 1.58E-02 |
| *LAMP3* | 3:182840000-182881627 | -4.68 | 1.45E-04 | 1.68E-02 |
| *AOAH* | 7:36550824-36764154 | -2.11 | 1.57E-04 | 1.78E-02 |
| *TNFSF15* | 9:117546931-117568718 | -2.15 | 1.60E-04 | 1.80E-02 |
| *PAQR5,Y_RNA* | 15:69591233-69740764 | 2.23 | 1.62E-04 | 1.82E-02 |
| *MIR221,MIR222* | X:45604650-45606530 | 2.09 | 1.62E-04 | 1.82E-02 |
| *MAP2K6,RP1-193H18.2* | 17:67410838-67550193 | -3.75 | 1.70E-04 | 1.90E-02 |
| *IQGAP2* | 5:75699073-76031606 | 2.06 | 2.14E-04 | 2.29E-02 |
| *CTA-392C11.2* | 8:40227546-40273606 | -4.88 | 2.49E-04 | 2.60E-02 |
| *AGAP11,BMS1P3,C10orf116,FAM25A,RP11-96C23.11* | 10:88695296-88784489 | -2.11 | 2.56E-04 | 2.67E-02 |
| *ROCK1P1* | 18:112114-120737 | -2.19 | 2.80E-04 | 2.85E-02 |
| *CTC-573N18.1* | 5:179868558-179870238 | -5.53 | 2.83E-04 | 2.88E-02 |
| *SNORA24* | 4:119199857-119200954 | -5.68 | 2.94E-04 | 2.95E-02 |
| *FAM189B* | 1:155216995-155225274 | 4.35 | 3.19E-04 | 3.14E-02 |
| *TRIM29* | 11:119981982-120056237 | -2.42 | 3.26E-04 | 3.19E-02 |
| *SNORD13* | 8:33221198-33371119 | 7.36 | 3.38E-04 | 3.28E-02 |
| *TMPRSS3* | 21:43791998-43816955 | -2.41 | 3.49E-04 | 3.37E-02 |
| *LCN2* | 9:130911349-130915734 | -3.04 | 3.58E-04 | 3.45E-02 |
| *D87015.1,IGLV2-14* | 22:23101184-23101707 | -6.41 | 4.43E-04 | 4.11E-02 |
| *KCNJ5* | 11:128759269-128792292 | 2.82 | 4.74E-04 | 4.36E-02 |
| *SLC30A10,ZC3H11B* | 1:219782858-220131989 | 2.78 | 4.73E-04 | 4.36E-02 |
| *RP5-1033H22.2* | 1:94027346-94312706 | -2.99 | 4.77E-04 | 4.38E-02 |
| *RNU6-16* | 11:88237743-88799113 | 7.18 | 5.36E-04 | 4.79E-02 |
| *LURAP1L* | 9:12685438-12823057 | -2.80 | 5.40E-04 | 4.81E-02 |
| *RP11-118B22.3* | 12:9381002-9428413 | -5.60 | 5.40E-04 | 4.81E-02 |
| *SNORD15B* | 11:75110529-75133324 | -6.53 | 5.64E-04 | 4.99E-02 |
