# Supplementary material for: The Genome-Wide Analysis of Carcinoembryonic Antigen Signaling by Colorectal Cancer Cells Using RNA Sequencing
Source: PLoS One. 2016 Sep 1;11(9):e0161256. doi: 10.1371/journal.pone.0161256 (PMC5008809; doi:10.1371/journal.pone.0161256)
Supplement: S2 Table — (DOCX) [file pone.0161256.s003.docx]

**S2_Table.xls.** **Functional enrichment of GO biological processes regulated by CEA expression level in MIP101 colorectal cancer cells**

| Gene Set Name | # Genes in Gene Set (K) | # Genes in Overlap (k) | k/K | p-value | FDR q-value |
| --- | --- | --- | --- | --- | --- |
| NEGATIVE_REGULATION_OF_CELLULAR_PROCESS | 646 | 12 | 0.0186 | 5.43E-09 | 2.20E-06 |
| CELL_PROLIFERATION_GO_0008283 | 513 | 11 | 0.0214 | 5.67E-09 | 2.20E-06 |
| NEGATIVE_REGULATION_OF_BIOLOGICAL_PROCESS | 677 | 12 | 0.0177 | 9.12E-09 | 2.20E-06 |
| REGULATION_OF_CELL_PROLIFERATION | 308 | 9 | 0.0292 | 1.07E-08 | 2.20E-06 |
| APOPTOSIS_GO | 431 | 9 | 0.0209 | 1.87E-07 | 2.47E-05 |
| PROGRAMMED_CELL_DEATH | 432 | 9 | 0.0208 | 1.90E-07 | 2.47E-05 |
| CELL_DEVELOPMENT | 577 | 10 | 0.0173 | 2.10E-07 | 2.47E-05 |
| POSITIVE_REGULATION_OF_CELL_PROLIFERATION | 149 | 6 | 0.0403 | 5.22E-07 | 5.39E-05 |
| NEGATIVE_REGULATION_OF_CELL_PROLIFERATION | 156 | 6 | 0.0385 | 6.84E-07 | 6.27E-05 |
| REGULATION_OF_APOPTOSIS | 341 | 7 | 0.0205 | 5.13E-06 | 3.92E-04 |
| REGULATION_OF_PROGRAMMED_CELL_DEATH | 342 | 7 | 0.0205 | 5.23E-06 | 3.92E-04 |
| RESPONSE_TO_STRESS | 508 | 8 | 0.0157 | 7.40E-06 | 5.09E-04 |
| EPIDERMIS_DEVELOPMENT | 71 | 4 | 0.0563 | 1.21E-05 | 7.67E-04 |
| ECTODERM_DEVELOPMENT | 80 | 4 | 0.05 | 1.94E-05 | 1.14E-03 |
| REGULATION_OF_DEVELOPMENTAL_PROCESS | 440 | 7 | 0.0159 | 2.65E-05 | 1.46E-03 |
| SYSTEM_DEVELOPMENT | 861 | 9 | 0.0105 | 4.98E-05 | 2.57E-03 |
| SIGNAL_TRANSDUCTION | 1634 | 12 | 0.0073 | 8.28E-05 | 4.02E-03 |
| TRANSCRIPTION | 753 | 8 | 0.0106 | 1.19E-04 | 5.46E-03 |
| TISSUE_DEVELOPMENT | 138 | 4 | 0.029 | 1.63E-04 | 6.37E-03 |
| NUCLEOTIDE_AND_NUCLEIC_ACID_METABOLIC_PROCESS | 1244 | 10 | 0.008 | 1.64E-04 | 6.37E-03 |
| POSITIVE_REGULATION_OF_EPITHELIAL_CELL_PROLIFERATION | 10 | 2 | 0.2 | 1.69E-04 | 6.37E-03 |
| ANATOMICAL_STRUCTURE_DEVELOPMENT | 1013 | 9 | 0.0089 | 1.70E-04 | 6.37E-03 |
| MULTICELLULAR_ORGANISMAL_DEVELOPMENT | 1049 | 9 | 0.0086 | 2.20E-04 | 7.89E-03 |
| RNA_METABOLIC_PROCESS | 841 | 8 | 0.0095 | 2.52E-04 | 8.43E-03 |
| TRANSCRIPTION_DNA_DEPENDENT | 636 | 7 | 0.011 | 2.61E-04 | 8.43E-03 |
| RNA_BIOSYNTHETIC_PROCESS | 638 | 7 | 0.011 | 2.66E-04 | 8.43E-03 |
| TISSUE_MORPHOGENESIS | 14 | 2 | 0.1429 | 3.40E-04 | 1.04E-02 |
| RESPONSE_TO_EXTERNAL_STIMULUS | 312 | 5 | 0.016 | 3.83E-04 | 1.12E-02 |
| RESPONSE_TO_CHEMICAL_STIMULUS | 314 | 5 | 0.0159 | 3.94E-04 | 1.12E-02 |
| BIOPOLYMER_METABOLIC_PROCESS | 1684 | 11 | 0.0065 | 4.53E-04 | 1.25E-02 |
| SENSORY_PERCEPTION | 190 | 4 | 0.0211 | 5.48E-04 | 1.46E-02 |
| RESPONSE_TO_ENDOGENOUS_STIMULUS | 200 | 4 | 0.02 | 6.64E-04 | 1.71E-02 |
| LOCOMOTORY_BEHAVIOR | 95 | 3 | 0.0316 | 8.83E-04 | 2.06E-02 |
| REGULATION_OF_TRANSCRIPTION | 566 | 6 | 0.0106 | 8.83E-04 | 2.06E-02 |
| REGULATION_OF_CELLULAR_METABOLIC_PROCESS | 787 | 7 | 0.0089 | 9.19E-04 | 2.06E-02 |
| NEUROLOGICAL_SYSTEM_PROCESS | 379 | 5 | 0.0132 | 9.20E-04 | 2.06E-02 |
| ORGAN_DEVELOPMENT | 571 | 6 | 0.0105 | 9.24E-04 | 2.06E-02 |
| NERVOUS_SYSTEM_DEVELOPMENT | 385 | 5 | 0.013 | 9.86E-04 | 2.12E-02 |
| REGULATION_OF_METABOLIC_PROCESS | 799 | 7 | 0.0088 | 1.00E-03 | 2.12E-02 |
| POSITIVE_REGULATION_OF_CELL_DIFFERENTIATION | 25 | 2 | 0.08 | 1.10E-03 | 2.28E-02 |
| CELL_CELL_SIGNALING | 404 | 5 | 0.0124 | 1.22E-03 | 2.46E-02 |
| REGULATION_OF_NUCLEOTIDE_AND_NUCLEIC_ACID_METABOLIC_PROCESS | 618 | 6 | 0.0097 | 1.38E-03 | 2.66E-02 |
| RESPONSE_TO_HYPOXIA | 28 | 2 | 0.0714 | 1.39E-03 | 2.66E-02 |
| REGULATION_OF_BIOLOGICAL_QUALITY | 419 | 5 | 0.0119 | 1.43E-03 | 2.69E-02 |
| TISSUE_REMODELING | 30 | 2 | 0.0667 | 1.59E-03 | 2.92E-02 |
| POSITIVE_REGULATION_OF_CELLULAR_PROCESS | 668 | 6 | 0.009 | 2.05E-03 | 3.65E-02 |
| TRANSCRIPTION_FROM_RNA_POLYMERASE_II_PROMOTER | 457 | 5 | 0.0109 | 2.09E-03 | 3.65E-02 |
| REGULATION_OF_GENE_EXPRESSION | 673 | 6 | 0.0089 | 2.12E-03 | 3.65E-02 |
| REGULATION_OF_TRANSCRIPTIONDNA_DEPENDENT | 461 | 5 | 0.0108 | 2.17E-03 | 3.66E-02 |
| REGULATION_OF_RNA_METABOLIC_PROCESS | 471 | 5 | 0.0106 | 2.38E-03 | 3.93E-02 |
