# Supplementary material for: The Genome-Wide Analysis of Carcinoembryonic Antigen Signaling by Colorectal Cancer Cells Using RNA Sequencing
Source: PLoS One. 2016 Sep 1;11(9):e0161256. doi: 10.1371/journal.pone.0161256 (PMC5008809; doi:10.1371/journal.pone.0161256)
Supplement: S3 Table — (DOCX) [file pone.0161256.s004.docx]

**S3_Table.xls. List of primers used for validation of RNAseq data by quantitative reverse transcriptase-polymerase chain reaction**

| gene | primer | sequence | Product-bp |
| --- | --- | --- | --- |
| *DSP* | 5’ | AGGCACCAGAACCAGAACAC | 184 |
|  | 3’ | TCAAGCTGTCGAGGATTTCCA |  |
| *PCDH1* | 5’ | ACCGTGCTTGACACCAATGA | 196 |
|  | 3’ | TGTCCAGTCGAAGAAGACGC |  |
| *WFS1* | 5’ | GGCGACACGGATGAAGAACT | 181 |
|  | 3’ | CCCTCTCCAGGTCGGTCTC |  |
| *GADD45A* | 5’ | TCCTGCTCTTGGAGACCGA | 161 |
|  | 3’ | ATCCATGTAGCGACTTTCCCG |  |
| *KLF11* | 5’ | TGCAGCCACACCTGAACTAC | 170 |
|  | 3’ | CTACGGCAGAGGACTGGAGA |  |
| *GAPDH* | 5’ | GGGAAGGTGAAGGTCGGAGT | 119 |
|  | 3’ | TTGAGGTCAATGAAGGGGTCA |  |
| *CEACAM5* | 5’ | GGCAACAGGACCCTCACTC | 195 |
|  | 3’ | GTGGCAGGAGAGGTTCAGATT |  |
